# Long Maximal Incremental Tests Accurately Assess Aerobic Fitness in Class II and III Obese Men

**DOI:** 10.1371/journal.pone.0124180

**Published:** 2015-04-13

**Authors:** Stefano Lanzi, Franco Codecasa, Mauro Cornacchia, Sabrina Maestrini, Paolo Capodaglio, Amelia Brunani, Paolo Fanari, Alberto Salvadori, Davide Malatesta

**Affiliations:** 1 Institute of Sport Sciences University of Lausanne (ISSUL), University of Lausanne, Lausanne, Switzerland; 2 Department of Physiology, Faculty of Biology and Medicine, University of Lausanne, Lausanne, Switzerland; 3 Pulmonary Rehabilitation Department, San Giuseppe Hospital, *Istituto Auxologico Italiano* Piancavallo, Verbania, Italy; 4 Molecolar Biology Laboratory, San Giuseppe Hospital, *Istituto Auxologico Italiano* Piancavallo, Verbania, Italy; 5 Orthopaedic Rehabilitation Unit and Clinical Lab for Gait and Posture Analysis, San Giuseppe Hospital, *Istituto Auxologico Italiano* Piancavallo, Verbania, Italy; 6 Medicine Rehabilitation Department, San Giuseppe Hospital, *Istituto Auxologico Italiano* Piancavallo, Verbania, Italy; Vanderbilt University, UNITED STATES

## Abstract

This study aimed to compare two different maximal incremental tests with different time durations [a maximal incremental ramp test with a short time duration (8-12 min) (S_Test_) and a maximal incremental test with a longer time duration (20-25 min) (L_Test_)] to investigate whether an L_Test_ accurately assesses aerobic fitness in class II and III obese men. Twenty obese men (BMI≥35 kg.m-2) without secondary pathologies (mean±SE; 36.7±1.9 yr; 41.8±0.7 kg*m^-2^) completed an S_Test_ (warm-up: 40 W; increment: 20 W*min-1) and an L_Test_ [warm-up: 20% of the peak power output (PPO) reached during the S_Test_; increment: 10% PPO every 5 min until 70% PPO was reached or until the respiratory exchange ratio reached 1.0, followed by 15 W.min^-1^ until exhaustion] on a cycle-ergometer to assess the peak oxygen uptake $\dot{V}O_{2peak}$ and peak heart rate (HR_peak_) of each test. There were no significant differences in $\dot{V}O_{2peak}$ (S_Test_: 3.1±0.1 L*min^-1^; L_Test_: 3.0±0.1 L*min^-1^) and HR_peak_ (S_Test_: 174±4 bpm; L_Test_: 173±4 bpm) between the two tests. Bland-Altman plot analyses showed good agreement and Pearson product-moment and intra-class correlation coefficients showed a strong correlation between $\dot{V}O_{2peak}$ (*r*=0.81 for both; *p*≤0.001) and HR_peak_ (*r*=0.95 for both; *p*≤0.001) during both tests. $\dot{V}O_{2peak}$ and HR_peak_ assessments were not compromised by test duration in class II and III obese men. Therefore, we suggest that the L_Test_ is a feasible test that accurately assesses aerobic fitness and may allow for the exercise intensity prescription and individualization that will lead to improved therapeutic approaches in treating obesity and severe obesity.

## Introduction

Maximal incremental exercise testing is commonly used in exercise physiology to determine physiological variables, such as peak oxygen uptake ($\dot{V}O_{2peak}$), peak heart rate (HR_peak_) and peak power output (PPO), together with other submaximal metabolic parameters [i.e., lactate (LT) and ventilatory thresholds (VT)]. The accuracy of the determination of these variables during a maximal incremental exercise test is important for exercise prescription and individualization in athletes, sedentary healthy individuals and patients [1]. It has been suggested that a maximal incremental exercise test should last between 8 and 12 minutes with short stage duration (1 min) to elicit $\dot{V}O_{2peak}$ [2–4], whereas longer protocols (~25 min) with long stage duration (3–5 min) report significantly lower $\dot{V}O_{2peak}$ [5–7] and PPO [8, 9] and higher HR_peak_ [5, 9, 10]. However, some studies report no significant differences in $\dot{V}O_{2peak}$ and HR_peak_ [8, 11–13], or in $\dot{V}O_{2}$ and HR at VT_1_ [7, 11], between short and long maximal incremental tests with different stage and time durations, suggesting that both exercises may have a practical relevance and may be useful in exercise intensity prescription and individualization in healthy men [14].

In class II and III obese individuals, exercise intensity prescription and individualization are strongly recommended as part of each patient’s multidisciplinary medical and surgical management in order to improve the poor aerobic fitness [15] and thus decrease the mortality risk in this population [16]. However, there are limited indications regarding which test is the most appropriate for the evaluation of aerobic fitness and the subsequent prescription of exercise training programs in class II and III obese individuals [15]. Severe obesity is also specifically characterized by a depressed capacity to oxidize lipids [17], which does not always occur at lower levels of obesity [18]. This decreased fat oxidation may be involved and contribute to the development of insulin resistance in severely obese individuals [17].

Endurance training targeting an exercise intensity (Fat_max_) that elicits the maximal fat oxidation (MFO) is appropriate in order to enhance fat oxidation rates and insulin sensitivity in obese individuals [19], highlighting the importance of correctly assessing Fat_max_. However, this is not possible with an incremental test with short stage duration, which is characterized by a non steady-state condition, but only with an incremental exercise test with longer stage duration (i.e., 5–6 min) during which steady state is reached for each step. Therefore, an incremental exercise test with longer stage duration may be an appropriate test to determine fat oxidation kinetics, MFO and Fat_max_ (metabolic fitness [20]) in obese and severely obese individuals [21]. Although it has previously been suggested that long test duration may affect $\dot{V}O_{2peak}$ assessment by reaching the limit of exercise tolerance earlier [3], this test has already been used to assess aerobic and metabolic fitness in class I and II obese individuals [22, 23]. In these studies, $\dot{V}O_{2peak}$seems to be correctly assessed because Fat_max_ (expressed in %$\dot{V}O_{2peak}$) has been found at similar values than those previously reported in obese subjects [19, 21, 24]. However, Ara et al. [22] and Larsen et al. [23] did not compare their maximal incremental long tests to a maximal incremental short test in order to attest whether long test elicits valid$\dot{V}O_{2peak}$.

Therefore, this study aimed to compare two maximal incremental tests with different time durations [a maximal incremental ramp test with a short time duration (8–12 min) (S_Test_) and a maximal incremental test with a longer time duration (20–25 min) (L_Test_)] in a group of class II and III obese men. It was hypothesized that the L_Test_ would elicit similar$\dot{V}O_{2peak}$, HR_peak_, $\dot{V}O_{2}$and HR at VT_1_ compared to the S_Test_, suggesting that the L_Test_ is an appropriate test to evaluate aerobic fitness. Moreover, this single test may also lead to simultaneously determine metabolic fitness (i.e., fat oxidation kinetics, MFO and Fat_max_) in order to obtain a more complete assessment of physical fitness in class II and III obese men and may aid in the exercise intensity prescription and individualization in this population.

## Materials and Methods

### Participants

Twenty obese men [body mass index (BMI)≥35 kg^.^m^-2^] without secondary pathologies were recruited to participate in this study (Table 1). Subjects were recruited from the *Istituto Auxologico Italiano* (Piancavallo, Italy). Subjects with hypertension [blood pressure (BP)>130/90 mmHg], impaired fasting glucose (>6.1 mmol^.^L^-1^) [25], type 2 diabetes and an abnormal electrocardiogram at rest were excluded. The study was approved by the Ethics Review Committee of the *Istituto Auxologico Italiano*, Italy. All subjects provided written, voluntary, informed consent before participating. The experiment was conducted according to the Declaration of Helsinki.

**Table 1:** Characteristics of the study subjects.

	Subjects
N	20
Age, yr	36.7 ± 1.9
Weight, kg	127.1 ± 3.4
Height, m	1.74 ± 0.02
BMI, kg^.^m^-2^	41.8 ± 0.7

Values are the means SE. BMI: body mass index.

### Experimental protocol

Subjects performed two maximal incremental tests to exhaustion on a cycle-ergometer (Ebike Basic BPlus, USA) to determine$\dot{V}O_{2peak}$, HR_peak_, peak ventilation (${\dot{V}}_{Epeak}$), peak respiratory exchange ratio (RER_peak_), PPO and VT_1_ ($\dot{V}O_{2}$, HR and PO) during each of the following tests: 1) a maximal incremental ramp test with a short time duration (8–12 min) (S_Test_) in the first session, and 2) a maximal incremental test with a longer time duration (20–25 min) (L_Test_) in the second session. This order was fixed because S_Test_ was necessary to individualise the warm-up and increments of the L_Test_ [21].

#### Maximal incremental ramp test with a short time duration (8–12 min) (S_Test_)

The S_Test_ was performed at least 2–3 h following the consumption of the last meal. After a 3-min rest period, subjects started with a 5-min warm-up at 40 W, after which the PO was linearly increased by 20 W every minute until exhaustion, which was determined by the inability to maintain a minimum pedalling frequency (i.e., 60 revolutions per min) despite verbal encouragement. This test was used previously [21] and yielded an exercise duration of approximately 10 min.

#### Maximal incremental test with a longer time duration (20–25 min) (L_Test_)

The L_Test_ was performed in the morning after a minimum of two days following the S_Test_. This test was performed in fasted state in order to determine the substrate oxidation. After a standardized 10-min warm-up at 20% PPO reached during S_Test_, the PO was increased by 10% PPO every 5 min until reaching 70% PPO, or until RER reached 1.0 (adapted from Lanzi et al. [21]). At this point, PO was increased by 15 W every minute until exhaustion as previously defined. From our previous data of a submaximal incremental test with 6 min stage duration [21], we determined that between the fourth and the fifth minute of each stage a steady-state condition was already reached in this population, therefore a protocol with 5 min stage was used to determine substrate oxidation and reduce test duration.

### Data analysis and calculation

#### Gas exchange

$\dot{V}O_{2}$, carbon dioxide production ($\dot{V}CO_{2}$) and ${\dot{V}}_{E}$ were measured continuously using a breath-by-breath online system (V_max_ 229, Sensor Medics, USA).$\dot{V}O_{2peak}$, ${\dot{V}}_{Epeak}$ and RER_peak_ were defined as the highest 10-s mean values recorded before the subject’s volitional termination of each test.

#### Peak heart rate and peak power output

HR was recorded continuously using an HR monitor (Polar RS800, Finland). HR_peak_ and PPO were defined as the highest peak values reached during each test.

#### Ventilatory threshold 1 and delta efficiency

VT_1_ ($\dot{V}O_{2}$, HR and PO) was determined during each test as described in the literature using Wasserman’s ventilatory method [26]. This method consists of visually determining the point at which the $\dot{V}O_{2}$ respiratory equivalent (${\dot{V}}_{E}$/$\dot{V}O_{2}$) increases as the $\dot{V}CO_{2}$ventilatory equivalent (${\dot{V}}_{E}$/$\dot{V}CO_{2}$) remains stable. The estimate of VT_1_ was supported using the Beaver ventilatory method [27]. This method consists of visually determining the inflection point of $\dot{V}CO_{2}$ with respect to$\dot{V}O_{2}$. Two blinded and independent investigators determined VT_1_. Delta efficiency (DE) was calculated as previously described [28].

#### Exercise intensity (Fat_max_) eliciting maximal fat oxidation

To determine if the L_Test_ is an accurate test to define Fat_max_ and to compare these results to previous findings, Fat_max_ was determined using the SIN model [29], as previously described in this population [21].

### Statistical analysis

Data are expressed as means±SE for all variables. Normal distribution of the variables was assessed using the Kolmogorov-Smirnoff test. Paired *t*-tests were used to compare peak and submaximal values between the two different maximal incremental exercise tests. To compare the agreement of the obtained peaks and VT_1_ values between the two different maximal incremental exercise tests, Bland–Altman plots were used [30]. The constructed graphs displayed scatter diagrams of the differences plotted against the mean of two measurements. The biases estimated from the mean differences ($\bar{m}$) were calculated, and 95% limits of agreement were estimated by $\bar{m}$±1.96 SD. To compare the agreement of the obtained peaks and VT_1_ values, we also assessed Pearson product-moment correlation and intra-class correlation (ICC) coefficients. The level of significance was set at *p*≤0.05.

## Results

### Characteristics of the tests

The duration of the L_Test_ was significantly longer (~2.6-fold) than the S_Test_ (23.2±0.5 and 8.8±0.3 min, respectively; *p*≤0.001). During the L_Test_, the mean warm-up load was 42±1 W, and the mean increment of the 5-min stage was 21±1 W.

### Peak exercise values

**Fig 1:**
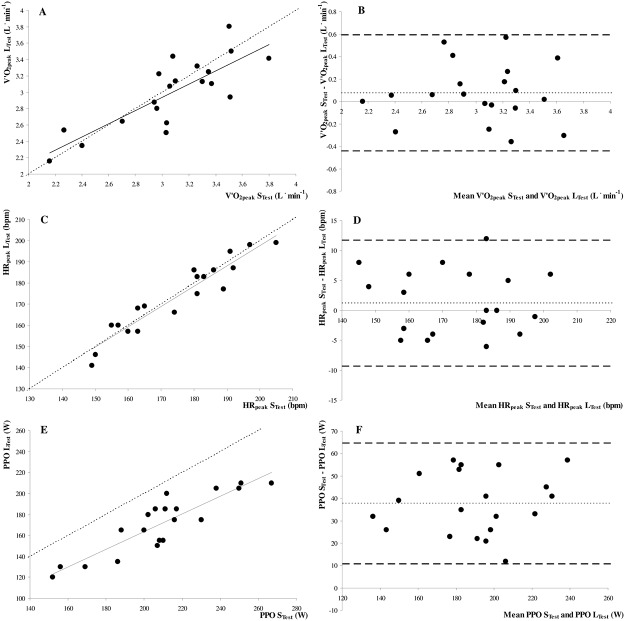
Correlations between A peak oxygen uptake ($\dot{V}O_{2peak}$; *y = 0*.*81x + 0*.*51*, *r* = 0.81, *p*≤0.001), C peak heart rate (HR_peak_; *y = 0*.*96x + 6*.*23; r* = 0.95, *p*≤0.001) and E peak power output (PPO; *y = 0*.*84x - 4*.*55; r* = 0.89, *p*≤0.001), and Bland-Altman plots of the absolute differences between B$\dot{V}O_{2peak}$, D HR_peak_ and F PPO determined during maximal incremental test with short (S_Test_) and long (L_Test_) time duration. In **A**, **C** and **E,** the dotted line represents the line of identity. In **B**, **D** and **F,** the light dotted line represents the bias from the mean difference, and the dark dotted line represents the upper and lower 95% limits of agreement.

$\dot{V}O_{2peak}$, HR_peak_ and ${\dot{V}}_{Epeak}$ were similar between the L_Test_ and S_Test_ (Table 2). By contrast, RER_peak_ and PPO were significantly lower in the L_Test_ than in the S_Test_ (Table 2). There was a strong correlation between $\dot{V}O_{2peak}$ (*r* = 0.81, *p*≤0.001; Fig 1A), HR_peak_ (*r* = 0.95, *p*≤0.001; Fig 1C) and ${\dot{V}}_{Epeak}$ (*r* = 0.67, *p* = 0.001; data not shown), as determined by the L_Test_ and S_Test_, and these data were close to the line of identity. RER_peak_ (*r* = 0.72, *p*≤0.001; data not shown) and PPO (*r* = 0.89, *p*≤0.001; Fig 1E) were also strongly correlated between the L_Test_ and S_Test_, although there was a systematic underestimation in the L_Test_ (i.e., data did not fit with the line of identity). These analyses were also confirmed by Bland–Altman plots (Fig 1B, 1D and 1F) and ICC analyses (Table 3). Biases and 95% limits of agreement for peak values between the L_Test_ and S_Test_ are shown in Table 3.

**Table 2:** Peak and ventilatory threshold 1 (VT_1_) values determined during the maximal incremental test with short (S_Test_) and long (L_Test_) time duration.

	S_Test_	L_Test_	*P* value
***Peak values***			
$\dot{V}O_{2peak}$, L^.^min^-1^	3.1 ± 0.1	3.0 ± 0.1	NS
HR_peak_, bpm	174 ± 4	173 ± 4	NS
${\dot{V}}_{Epeak}$, L^.^min^-1^	118.9 ± 4.2	115.8 ± 5.4	NS
RER_peak_	1.11 ± 0.01	1.00 ± 0.01	≤0.001
PPO, W	209 ± 7	171 ± 6	≤0.001
***VT***_***1***_ ***values***			
$\dot{V}O_{2}$, L^.^min^-1^	1.6 ± 0.0	1.6 ± 0.0	NS
HR, bpm	126 ± 2	116 ± 2	≤0.001
PO, W	103 ± 4	81 ± 4	≤0.001

Values are the means SE.$\dot{V}O_{2peak}$: peak oxygen uptake; HR_peak_: peak heart rate;${\dot{V}}_{Epeak}$: peak ventilation; RER_peak_: peak respiratory exchange ratio; PPO: peak power output; NS: non significant.

**Table 3:** Intra-class correlation, biases and 95% limit of agreement of the peak and ventilatory threshold 1 (VT_1_) values between the maximal incremental test with short (S_Test_) and long (L_Test_) time duration.

	Intra-class correlation (ICC)	Bias (IC)	Upper limit of agreement	Lower limit of agreement
***Peak values***				
$\dot{V}O_{2peak}$, L^.^min^-1^	0.81*	0.07 (± 0.12)	0.59	-0.44
HR_peak_, bpm	0.95*	1.20 (± 2.36)	11.74	-9.34
${\dot{V}}_{Epeak}$, L^.^min^-1^	0.66*	3.14 (± 7.88)	38.36	-32.09
RER_peak_	0.23*	0.12 (± 0.02)	0.20	0.03
PPO, W	0.48*	37.80 (± 6.03)	64.78	10.82
***VT***_***1***_ ***values***				
$\dot{V}O_{2}$, L^.^min^-1^	0.69*	0.07 (± 0.07)	0.36	-0.23
HR, bpm	0.47*	9.42 (± 3.64)	25.31	-6.47
PO, W	0.44*	21.25 (± 5.88)	47.55	-5.05

Values of bias are the means ± interval confidence (IC). Biases and 95% limits of agreements were estimated with Bland–Altman method.$\dot{V}O_{2peak}$: peak oxygen uptake; HR_peak_: peak heart rate;${\dot{V}}_{Epeak}$: peak ventilation; RER_peak_: peak respiratory exchange ratio; PPO: peak power output.

* *p*≤0.05 for significant ICC coefficient.

### Ventilatory threshold and delta efficiency values

**Fig 2:**
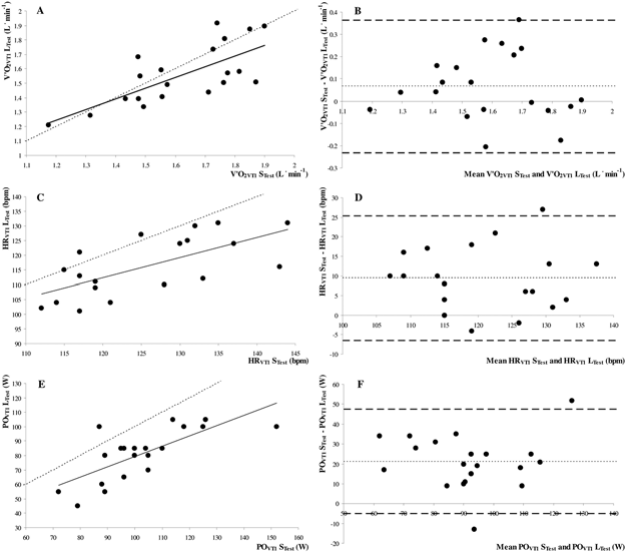
Correlations between A oxygen uptake at ventilatory threshold 1 ($\dot{V}O_{2VT1}$; *y = 0*.*75x + 0*.*35; r* = 0.72, *p*≤0.001), C heart rate at VT_1_ (HR_VT1_; *y = 0*.*69x + 29*.*98; r* = 0.67, *p* = 0.001) and E power output at VT_1_ (PO_VT1_; *y = 0*.*71x + 8*.*21; r* = 0.73, *p*≤0.001), and Bland-Altman plots of the absolute differences between B$\dot{V}O_{2VT1}$, D HR_VT1_ and F PO_VT1_ determined during maximal incremental test with short (S_Test_) and long (L_Test_) time duration. In **A**, **C** and **E,** the dotted line represents the line of identity. In **B**, **D** and **F,** the light dotted line represents the bias from the mean difference, and the dark dotted line represents the upper and lower 95% limits of agreement.

$\dot{V}O_{2VT1}$was similar between the L_Test_ and S_Test_ (Table 2). By contrast, HR_VT1_ and PO_VT1_ were significantly lower in the L_Test_ than in the S_Test_ (Table 2). There was a strong correlation between the $\dot{V}O_{2VT1}$ (*r* = 0.72, *p*≤0.001; Fig 2A), as determined by the L_Test_ and S_Test_, and these data were close to the line of identity. HR_VT1_ (*r* = 0.67, *p* = 0.001; Fig 2C) and PO_VT1_ (*r* = 0.73, *p*≤0.001; Fig 2E) were strongly correlated between the L_Test_ and S_Test_, although there was an underestimation in the L_Test_ (i.e., data did not fit with the line of identity). These analyses were also confirmed by Bland–Altman plots (Fig 2B, 2D and 2F) and ICC analyses (Table 3). Biases and 95% limits of agreement for VT_1_ values between the L_Test_ and S_Test_ are shown in Table 3. DE was lower during L_Test_ than during S_Test_ (17.8±0.5 and 22.5±0.5%, respectively; *p*≤0.001).

### Exercise intensity eliciting maximal fat oxidation

The Fat_max_ during the L_Test_ was found at 50.6±1.9%$\dot{V}O_{2peak}$.

## Discussion

The results of this study showed that$\dot{V}O_{2peak}$, HR_peak_ and $\dot{V}O_{2VT1}$ assessments were not compromised by prolonged stage and test duration, suggesting that the L_Test_ is an appropriate test for evaluating aerobic fitness and may be used for prescribing an exercise training regimen in class II and III obese men. There was, however, a significant influence exerted by time duration on PPO, HR and PO at VT_1_.

### Oxygen uptake

The data of the present investigation show that $\dot{V}O_{2peak}$ was statistically similar and showed good agreement between the L_Test_ and S_Test_ (correlation coefficients and Bland–Altman plot analyses). These results are in line with previous studies, which reported a similar $\dot{V}O_{2peak}$ between short (~10 min) and long (~25 min) maximal incremental tests with different stage and time durations [8, 9, 11] in healthy normal-weight individuals, suggesting that the dogmatic view that maximal incremental tests should last between 8 and 12 min to elicit $\dot{V}O_{2peak}$ [2–4] should be reconsidered [14]. Additionally, $\dot{V}O_{2VT1}$also showed good agreement with respect to the L_Test_ and S_Test_, and these results are in line with previous studies that showed that $\dot{V}O_{2VT1}$ was independent of exercise test duration [3, 7]. However, our results contrast with previous studies that reported different $\dot{V}O_{2peak}$ between short and long maximal incremental tests [3, 4, 7]. The reason for this discrepancy is unclear but may be due to different factors, such as different exercise test protocols (e.g., stage *vs*. ramp increments). Furthermore, previous studies compared normal and highly trained subjects, whereas this is the first study comparing individuals with a high degree of obesity (BMI ≥ 35 kg^.^m^-2^). The Bland–Altman plot analysis of $\dot{V}O_{2peak}$ was similar to previous studies, which reported a mean bias of 0.1 L^.^min^-1^ [8, 9], with 95% limits of agreement between 0.4 and -0.6 L^.^min^-1^ [8] (which was considered good agreement) between short and long maximal incremental tests with different stage and time durations in well trained triathletes. However, for some individuals (n = 3), the difference in $\dot{V}O_{2peak}$ between the S_Test_ and L_Test_ was greater (0.41, 0.53 and 0.57 L^.^min^-1^) than the mean bias (Fig 1A and 1B). This result suggests that these subjects presented with consistently lower $\dot{V}O_{2peak}$ during the L_Test_ compared to the S_Test_. Interestingly, these three individuals completed only one or two stages of 15 W^.^min^-1^ increments after having completed four stages of 5 min (i.e., 30, 40, 50 and 60% PPO reached during the S_Test_), whereas the other subjects completed up to five 5-min steps (until 70% PPO) or as many as five 1-min steps. It is therefore possible that a premature fatigue state of some subjects may explain the lower $\dot{V}O_{2peak}$ obtained during the L_Test_ [8], suggesting that envisaging a 5-min rest before starting increments of 15 W^.^min^-1^ during the L_Test_ may be a reasonable approach of eliciting$\dot{V}O_{2peak}$, as previously described [22].

### Heart rate

HR_peak_ was also statistically similar and showed very good agreement between the L_Test_ and S_Test_. Although some studies reported higher HR_peak_ during prolonged incremental exercise tests [5, 9, 10] (most likely linked to higher body temperatures or increased skin blood flow compared to parameters observed during short incremental exercise tests [3]), other studies suggested that HR_peak_ may not be affected by stage and exercise test duration [8, 11–13, 31]. Additionally, our results are similar to others [8], who reported a mean bias of 3 bpm, with 95% limits of agreement between 6 and -12 bpm between short and long maximal incremental tests with different stage durations in well trained triathletes. On the other hand, contrary to Weston et al. [7], HR_VT1_ was lower during the L_Test_ compared to the S_Test_. However, the HR_VT1_ mean bias was ~9 bpm (~5%) between the two tests, and it may be within the range of day-to-day HR variability [32]; therefore, it may be useful in prescribing an appropriate training regimen.

### Power output

In line with previous studies [7–9], our results show the significant influence of protocol time duration on PPO, findings similar to those of Bishop et al. [9], who reported a mean bias of 34.4 W, with 95% limits of agreement between 59.7 and 9.0 W between short and long maximal incremental tests with different stage and time durations in moderately active females. Interestingly, the results of the present study and those of Bishop et al. [9] show that PPO demonstrated good correlations with respect to short and long maximal incremental tests, although a systematic underestimation of PPO in prolonged exercise was noted (Fig 1E), also attested by lower ICC coefficient. Similarly, as previously reported [7], PO_VT1_ was also significantly lower during the L_Test_. The higher PO_VT1_ noted during the S_Test_ may be related to the physiological lag time between the increase in work rate and gas exchange responses, leading to an overestimation of VT_1_ when expressed as a work rate (PO_VT1_) but not when expressed as metabolic units ($\dot{V}O_{2VT1}$) [7]. Moreover, although not measured, it is possible that the higher PPO observed during the S_Test_ was related to lower blood lactate concentrations during the S_Test_ compared to the L_Test_, allowing subjects to attain a higher PO before suffering from local muscle fatigue [7, 11]. Additionally, the $\dot{V}O_{2}$ slow component for exercises above the VT_1_ [33] may be undetectable until the end of testing during rapidly-incremental ramp tests [34] but has sufficient time to be expressed during prolonged exercise tests [35], which may explain the lower PPO but similar $\dot{V}O_{2peak}$ and the lower DE noted during the L_Test_.

### L_Test_ and exercise training prescriptions

It has been established that monitoring $\dot{V}O_{2}$ and HR during effort is the most commonly used method of prescribing and individualizing exercise training to determine exercise intensity (expressed in %$\dot{V}O_{2peak}$ and %HR_peak_). Moreover, training target zones are also usually defined based on %$\dot{V}O_{2peak}$ and %HR_peak_ to individualize exercise training regimens and to determine the effects of a training session [32, 36]. In obese individuals, the *individualization concept of training* plays a pivotal role in weight management, particularly in reducing cardiovascular risk and the risk of developing secondary pathologies [37]. Indeed, it has been demonstrated that various forms of training for which exercise intensity was individualized at a target %HR_peak_ (corresponding to VT_1_ [38], moderate intensities [39, 40] and high-intensities [40–42]) determined by a short (~10 min) maximal incremental test may improve health-related outcomes (i.e., $\dot{V}O_{2peak}$, muscle oxidative capacity, lipid profiles and insulin sensitivity) in this population. From a clinical standpoint, as our results show good agreement in HR and $\dot{V}O_{2}$ between the L_Test_ and S_Test_: we believe that the L_Test_ is also an appropriate test for evaluating aerobic fitness and for prescribing exercise training regimens in class II and III obese men. Additionally, compared to short incremental tests, prolonged incremental exercise may also be used to assess fat oxidation kinetics, MFO and Fat_max_ in obese and severely obese individuals [21]. Indeed, it has been previously demonstrated that individualized Fat_max_ training may significantly increase muscle oxidative capacity, as well as fat oxidation rates during exercise and insulin sensitivity in obese individuals [19, 43], highlighting its clinical relevance in the treatment of obesity [37] and the importance of correctly assessing Fat_max_ as a function of measured $\dot{V}O_{2peak}$ [44]. However, to reduce the number of times that subjects have to report to the laboratory before starting training, it is preferable that only one test be performed. Therefore, we suggest that a prolonged incremental exercise test that starts with a 10-min warm-up at 40 W, followed by 20 W increments every 5 min until reaching 120–140 W (i.e., 4 or 5 stages), followed by 15 W increments every minute until exhaustion would be a feasible and accurate test for assessing aerobic fitness and prescribing an exercise training regimen in class II and III obese men.

### Methodological considerations

Some methodological limitations arose from the study and need to be further addressed. Firstly, the subjects always completed the S_Test_ first and the L_Test_ second. Although a randomised counterbalanced test order would have been preferable, in our study design we need to firstly conduct the S_Test_ with regard to determine the correct PO for the warm-up and for the 5-min stage increments during the L_Test_ in order to individualise each protocol and obtain enough points to assess fat oxidation kinetics, MFO and Fat_max_ in our subjects [29]. Moreover, through this study design, we were able to develop a single test protocol specific to class II and III obese men that accurately and simultaneously assess aerobic and metabolic fitness (see above for details). In this line, Fat_max_ seems to be accurately assessed during L_Test_ because has been found at similar values (~51%$\dot{V}O_{2peak}$) than those previously reported in this population [19, 21–24]. However, further investigations are needed to confirm this claim. Secondly, as we focused primarily on $\dot{V}O_{2peak}$ and not on$\dot{V}O_{2\max}$, our results may also have been affected. However, it was recently suggested that $\dot{V}O_{2peak}$ may also be indicative of a true $\dot{V}O_{2\max}$ in both lean [45] and obese individuals [1]. Additionally, previous studies have already compared $\dot{V}O_{2peak}$ between two different maximal incremental tests with different stage and test durations in normal-weight individuals [7–9, 11]. Moreover, the observed agreement in HR_peak_ and $\dot{V}O_{2peak}$ with respect to the L_Test_ and S_Test_ suggests that these measurements are reproducible with different tests in class II and III obese men. However, the lower RER obtained during the L_Test_ may be related to the depletion of bicarbonate reserves as a result of increased time spent above VT_1_ [10], suggesting that the use of RER as an indicator of maximal effort in the setting of prolonged incremental tests should be reconsidered.

In summary, we demonstrate that$\dot{V}O_{2peak}$, HR_peak_ and $\dot{V}O_{2VT1}$ assessments were not compromised by prolonged test durations in class II and III obese men. Therefore, we suggest that the L_Test_ is a feasible and accurate maximal incremental test and may be used to evaluate aerobic and metabolic fitness and to prescribe exercise training regimens to improve therapeutic approaches used to treat obesity and severe obesity.
